# LncRNA SNHG14 Delivered by Bone Marrow Mesenchymal Stem Cells‐Secreted Exosomes Regulates Osteogenesis and Adipogenesis in Osteoporosis by Mediating the miR‐27a‐3p/LMNB1 Axis

**DOI:** 10.1002/kjm2.70004

**Published:** 2025-03-07

**Authors:** Jin‐Shan Tang, Huai‐Xi Yu, Ru‐Xin Ruan, Rui Chen, Zi‐Qiang Zhu

**Affiliations:** ^1^ Department of Joint Surgery Huai'an Second People's Hospital (The Affiliated Huaian Hospital of Xuzhou Medical University) Huai'an City Jiangsu Province China; ^2^ Department of Orthopaedics The Second Affiliated Hospital of Xuzhou Medical University Xuzhou City Jiangsu Province China

**Keywords:** adipogenesis, bone marrow mesenchymal stem cells, exosomes, LncRNA SNHG14, osteogenesis

## Abstract

The purpose of this study was to investigate the role of LncRNA SNHG14 delivered by bone marrow mesenchymal stem cells‐secreted exosomes (BMSC‐Exos) in osteoporosis (OP). BMSCs and BMSCs‐Exos were isolated and identified. BMSCs were transfected, from which BMSCs‐Exos were collected. The treated BMSCs‐Exos were co‐cultured with BMSCs. After osteogenic induction of BMSCs, the calcification was analyzed by alizarin red S staining. After adipogenic induction of BMSCs, lipid droplets were detected by oil red O staining. Glycerol‐3‐phosphate dehydrogenase activity was measured in BMSCs. OVX mouse models were established and treated with BMSC‐EXOs. HE staining and IHC staining were performed on the femurs of mice. The interaction between SNHG14, miR‐27a‐3p, and LMNB1 was evaluated by luciferase reporter gene assay and RIP assay. Gene levels were assessed using RT‐qPCR and Western blot, respectively. BMSC‐Exos promoted osteogenic‐adipogenic balance of BMSCs. SNHG14 enhanced the promoting effect of BMSCs‐Exos on the balance of osteogenesis and adipogenesis of BMSCs. SNHG14 directly bound miR‐27a‐3p. SNHG1 mediated osteogenic‐adipogenic balance in BMSCs via miR‐27a‐3p. LMNB1 was a target gene of miR‐27a‐3p. LMNB1 was involved in the process of SNHG14 regulating osteogenic‐adipogenic balance in BMSCs. SNHG14 overexpression promoted bone formation and alleviated OP in vivo. SNHG14 delivered by BMSCs‐Exos regulates osteogenesis and adipogenesis in OP by mediating the miR‐27a‐3p/LMNB1 axis.

## 1 Introduction

Individuals in their middle and later years are afflicted with osteoporosis (OP), a persistent bone metabolic disorder linked to significant illness and impairment, prolonged disease duration, and elevated medical expenses. Estimates suggest that half of the global postmenopausal female population suffers from this illness, with about 40% of them eventually developing osteoporotic fractures [1, 2]. The majority of market‐available medications primarily focus on bone balance processes mediated by osteoblasts and osteoclasts, utilizing bisphosphonates and estrogen receptor drugs. Nonetheless, these medications come with disadvantages, such as poor absorption and negative side effects, leading to unsuccessful treatments [3]. Bone marrow mesenchymal stem cells (BMSCs) are multifunctional stem cells capable of differentiating into osteoblasts, osteoclasts, and adipocytes [4]. The differentiation capacity of BMSCs has been reported to be associated with OP. Specifically, reduced osteogenic differentiation of BMSCs causes disturbances in bone formation and promotes OP [5]. Therefore, BMSCs‐based therapy may serve as a breakthrough in the treatment of OP, and somehow promoting the osteogenic differentiation of BMSCs will help alleviate OP.

OP is associated with biochemical alterations in BMSCs, including reduced osteoblast numbers and differentiation ability, increased apoptosis, or decreased adipogenesis [6, 7]. A contradictory link exists between osteogenic and adipogenic differentiation. In the bone marrow of OP patients, there is a significant increase in adipocytes that corresponds with a decrease in bone mass, a phenomenon known as bone‐lipid balance [8]. More importantly, cytokines associated with osteogenic differentiation are secreted by adipocytes [9]. These cytokines influence the differentiation of BMSCs and regulate osteogenic and adipogenic markers [10]. Exosomes derived from osteoblasts regulate osteoclast activity and bone metabolism in bone microenvironment [11]. BMSCs‐Exosomes (BMSCs‐Exos) are a type of BMSCs‐derived extracellular vesicles with roles in intercellular communication, tissue repair, and immunomodulation, and may regulate the function of BMSCs themselves through autocrine effects. BMSCs‐Exos attenuate radiation‐induced bone loss [12]. Therefore, OP may benefit from controlling the balance between osteogenesis and adipogenesis.

Long‐chain non‐coding RNAs (lncRNAs) are emerging as novel regulators of osteogenesis in mesenchymal stem cell (MSCs) [13]. Various lncRNAs are involved in osteoblast differentiation of BMSCs, such as lncRNA‐ANCR [14] and lncRNA H19 [15]. Currently, it is known that SNHG14 induces osteogenic differentiation of BMSCs in vitro [16], there is, however, no clear indication that exosomal SNHG14 plays a role in OP development.

A critical role for miRNAs is to regulate bone remodeling, especially bone structure to balance bone formation, bone resorption, and OP [17]. miR‐27a‐3p has been found to induce osteogenic differentiation in BMSCs through the activation of osteogenic genes [18]. Therefore, the application of miR‐27a‐3p in OP therapy is of interest and requires further study. Lamin B1 (LMNB1) is a key B‐type nuclear fibrillar layer protein that regulates apoptosis and functional signals in tumorigenesis [19]. Zhang et al. performed protein expression profiling using quantitative proteomics in 42 postmenopausal women with inconsistent levels of bone mineral density (BMD) and demonstrated that LMNB1 was up‐regulated in patients with low BMD [20].

The aim of this study was to investigate the role of exosomal SNHG14 in BMSCs in OP, clarify the possible regulatory mechanisms of exosomal SNHG14 with miR‐27a‐3p and LMNB1, and provide promising targets and diagnostic biomarkers for OP.

## 2 Materials and Methods

### 2.1 Isolation and Culture of BMSCs

Primary BMSCs were obtained from the femur and tibia of mice. Bones were dissected in a sterile environment, and total bone marrow was obtained by flushing the marrow cavity with DMEM (Gibco, USA). BMSCs were cultured to passages 3–5 and centrifuged at 1000 × *g* for 5 min.

The identification of BMSCs utilized the MSC Surface Labeling Assay Kit (RAXMX‐09011). BMSCs were dispersed in 1 × PBS with 0.1% bovine serum albumin, combined with trypan blue in equal parts, and tallied using a Shanghai Ruiyu IC1000 automatic cell counter. After modifying the cell density to 3 × 10^6^ cells/mL, BMSCs underwent a 30‐min incubation at 4°C with CD44 (Cyagen Biosciences, CA, USA) and CD45 (Cyagen Biosciences). The fluorescent secondary antibody FITC (Cyagen Biosciences) was introduced at 4°C for half an hour, and the proportion of positive cells was examined using a NovoCyte flow cytometer (ACEA Biosciences, CA, USA) along with the NovoExpressTM software (NovoCyte, CA, USA).

BMSCs were cultured in DMEM supplemented with 10% FBS (Gibco), 1% penicillin and streptomycin (Gibco), and the culture medium was changed every 3–4 days.

To induce osteogenic differentiation, cells at 70% confluence were cultured in an osteogenic induction medium (HUXMA‐90021, Cyagen Biosciences) containing 10 mM β‐glycerophosphate (G9422), 0.2 mM ascorbic acid (A8960), 10–5 mM dexamethasone (D4902) (all from Sigma‐Aldrich), and 10 mM 1.25‐vitamin‐D3 for 3 days. The medium was changed every 3 days. Cells were collected 2–3 weeks after induction.

To induce adipogenic differentiation, cells at 100% confluence were cultured in an adipogenic induction medium A solution (MUD‐90031, Sailing, China) containing 1 μM dexamethasone, 10 μg/mL insulin, 0.5 mM 3‐isobutyl‐1‐methylxanthopterin, and 100 μM indomethacin for 3 days. The medium was replaced with AIM B solution (Cyagen Biosciences, China) after incubation for 1 day. The induction lasted for 16 days with 4 days as a cycle.

### 2.2 Isolation and Characterization of BMSC‐Exos

After BMSCs were grown to 80%–90% confluence, they were placed in a serum‐free DMEM for 48 h. The supernatant was first centrifuged at 300 × *g* for 10 min and then at 2000 × *g* for 10 min. The supernatant was centrifuged at 10,000 × *g* for 30 min using an Optima XPN‐100 high‐speed centrifuge (CR21G, Hitachi, Japan) to remove cellular debris, which was passed through a 0.22‐μm filter to remove larger particles. The supernatant was then centrifuged at 100,000 × *g* for 70 min in an ultra‐high speed centrifuge (CP100NX, Hitachi) and at 110,000 × *g* for 70 min. BMSC‐Exos were resuspended in PBS (1 μL PBS/1 mL supernatant). It was performed at 4°C for all steps.

The morphology of BMSC‐Exos was observed under a 100 kV transmission electron microscope (Hitachi, H‐7000FA). BMSC‐Exos‐specific biomarkers CD9 (A1703, Abclonal, Wuhan, China), CD63 (A5271, Abclonal), and TSG101 (A5789, Abclonal) were detected by Western Blot.

### 2.3 BMSC‐Exos Labeling and Uptake

BMSC‐Exos underwent staining using the PKH67 kit (BestBio, China), were passed through a 0.22 μm filter, and then dissolved in sterile PBS. BMSCs were treated with PKH67‐tagged BMSC‐Exos and grown in a serum‐free medium for a day. Subsequently, BMSCs were stabilized using 4% paraformaldehyde for half an hour, dyed with 4′,6‐diamidino‐2‐phenylindole for 10 min, and visualized using a fluorescence microscope (Leica, UK).

### 2.4 Nanoparticle Tracking Analysis (NTA)

A total of 20 μg of BMSC‐Exos was dissolved in 1‐mL PBS and spun for 1 min. The samples were used to measure the diameter size with NTA (Malvern Panalytical, Worcestershire, UK). The particles were automatically tracked and sized according to Brownian movement and diffusion coefficients. The measurement conditions were 21.0°C ± 0.5°C, 0.99°Cp, 25 frames/s, and 30 s measurement time. The detection threshold was the same for all samples. Each sample was tested three times.

### 2.5 Cell Transfection

The 3rd passage BMSCs were digested by trypsin (Beyotime) and cultured in 24‐well plates (1 × 10^6^ cells/well), and when 85% cell confluence was reached, transfection was achieved in BMSCs by Lipofectamine 3000 (Thermo Fisher Scientific). oe‐SNHG14, oe‐NC, miR‐27a‐3p mimic, miR‐NC, sh‐NC, and sh‐LMNB1 were synthesized by GeneChem (Shanghai, China). Forty‐eight hours after transfection, supernatants from BMSCs were harvested to isolate exosomes, which were co‐cultured with BMSCs after treatment as indicated.

### 2.6 Dual Luciferase Assay

Potential binding sites for miR‐27a‐3p and SNHG14 or LMNB1 were predicted by starBase 3.0 (http://starbase.sysu.edu.cn/). Synthesized wild‐type or mutant SNHG14 or LMNB1 fragments (GenePharma, Shanghai, China) containing miR‐27a‐3p binding sites were cloned into pmirGLO luciferase reporters (Promega, WI, USA) to produce SNHG14‐WT, LMNB1‐WT, SNHG14‐MUT, and LMNB1‐MUT. BMSCs were put in 96‐well plates at 1 × 10^4^ cells/well and transfected with luciferase reporter and miR‐27a‐3p mimic or mimic‐NC using Lipofectamine 3000 (Invitrogen), and the culture was continued for 48 h. The luciferase activity was detected by the Dual Luciferase Assay System (Promega).

### 2.7 RIP Experiments

RIP assays were performed using the Magna RIP Kit (Millipore). BMSCs were lysed with RIP lysis buffer, and the cell lysates (10 μL) were co‐incubated with magnetic beads containing anti‐Ago2 (Abcam, MA, USA) or anti‐IgG (Abcam) for 6 h at 4°C. Immunoprecipitates bound to magnetic beads were eluted, and SNHG14, miR‐27a‐3p, and LMNB1 were analyzed by RT‐qPCR after RNA purification.

### 2.8 RT‐qPCR

Total RNA was extracted from tissues and BMSCs using Trizol reagent (Thermo) and RNA concentration was measured using a BCA kit (Invitrogen). miRNA reverse transcription kit (TaKaRa) was used for cDNA synthesis of miRNAs, and cDNAs for LncRNAs and mRNA were synthesized using the PrimeScript RT Reagent kit (TaKaRa). PCR was conducted using the SYBR green PCR premix kit (Invitrogen) on a CFX96 contact real‐time fluorescent quantitative PCR detection system (Bio‐Rad, CA, USA). The primer sequences are shown in Table 1, with the values of RNA levels calculated using the 2^−ΔΔCt^ method.

**TABLE 1:** Primer sequence.

Gene	Forward primer (5′ → 3′)	Reverse primer (5′ → 3′)
LncRNA SNHG14	GGGTGTTTACGTAGACCAGAACC	CTTCCAAAAGCCTTCTGCCTTAG
miR‐27a‐3p	GCGCATTCACAGTGGCTAAG	GTCGTATCCAGTGCAGGGTCCGAGGTATTCGCACTGGATACGACGCGGAA
LMNB1	AAGCAGCTGGAGTGGTTGTT	TTGGATGCTCTTGGGGTTC
ALP	GGGCAATGAGGTCACATCCA	GTGGTTCACCCGAGTGGTAG
GAPDH	AGCCCAAGATGCCCTTCAGT	CCGTGTTCCTACCCCCAATG

### 2.9 Western Blot

BMSCs and tissues were lysed on ice for 20 min using RIPA lysis buffer (Vazyme, FD008). Protein concentration was assayed using the Pierce ALI Protein Assay Kit (Rockford). Proteins were separated using 10% SDS‐PAGE and transferred to PVDF membranes (Millipore). The membranes were blocked with 5% skimmed milk for 2 h, incubated with primary antibodies against LMNB1 (AmyJet Scientific; PAB12282), GAPDH (Abcam; ab37168), Runx2 (Abcam; ab236639), OCN (Abcam; ab93876), PPARγ (Abcam; ab209350), and C/EBPα (AmyJet Scientific; ABP50804) overnight at 4°C and with the secondary antibody (Abcam; ab205719) for 1 h at 37°C. Finally, the results were visualized with the Enhanced Chemiluminescence Detection Kit (E411‐04, Vazyme) and checked on the FluorChemM system.

### 2.10 Alizarin Red S Staining

Post osteogenic induction, BMSCs were fixed with 95% ethanol for 10 min and subsequently stained using the alizarin red kit (G1450, Solarbio, Beijing, China). Observations of the dyed calcium nodules were conducted using a light microscope (OLYMPUS, Tokyo, Japan).

### 2.11 Oil Red O Staining

Post adipogenic differentiation, BMSCs underwent fixation using 10% neutral formalin for an hour, followed by staining with the Oil Red O Staining Kit (G1262, Solarbio), rinsing with distilled water, and examination under an inverted microscope (Leica).

### 2.12 Glycerol‐3‐Phosphate Dehydrogenase (G‐3‐PDH) Measurement

Since G‐3‐PDH is specifically present in mature adipocytes, its activity was gauged to monitor triglyceride production in BMSCs. In each well, BMSCs were mixed with 0.5 mL of lysis buffer (comprising 50 mM Tris, 0.1 mM β‐mercaptoethanol, and 1 mM EDTA) and subjected to 30 s of sonication in ice. Subsequently, 300 μL of this mixture was combined with 1.5 mL of assay buffer (comprising, 2.5 mM EDTA, 100 mM triethanolamine hydrochloride, 0.1 mM β‐mercaptoethanol, and 0.12 mM NADH), and the activity of G‐3‐PDH was assessed using a 340 nm UV spectrophotometer. The addition of 100 μL of Dihydroxyacetone phosphate buffer was followed by a reassessment of its activity at 340 nm.

### 2.13 Establishment of Ovariectomy (OVX) Mouse Model

Twenty adult female BALB/c mice (9 weeks old, 18–22 g) were purchased from Hunan Experimental Animal Center, and were randomly divided into (1) the Sham group; (2) the OVX group; (3) the OVX + EXOs‐oe‐NC; (4) the OVX + EXOs‐oe‐ SNHG14 group (*n* = 5). Mice were anesthetized with 30 mg/kg pentobarbital sodium, and two bilateral incisions of 10 mm diameter were made in the skin on the lumbar side. The dorsal mid‐back region of each mouse was shaved. The shaved skin was cleaned with 70% ethanol. A midline dorsolateral skin incision was made, and the bilateral ovaries of the mice were carefully excised. All mice in the control group underwent the same procedure without removing the bilateral ovaries. The tissues were then repositioned and sutured. Mice were injected with 40,000 IU/mL penicillin at 1 mL/kg for 3 days. This was continued for 2 months. Mice were subjected to 28 consecutive days of pharmacological intervention and administered as follows: 100 μL BMSC‐EXOs (10^13^/mL) harvested after transfection with oe‐SNHG14 or oe‐NC were injected into OVX mice by tail vein once a week. After 28 days, mice were euthanized by intraperitoneal injection with pentobarbital sodium at 250 mg/kg, serum from each group was collected, and femoral tissue was isolated under sterile conditions for subsequent experiments.

### 2.14 BMD and Hematoxylin–Eosin (HE) Staining

Femurs were harvested for BMD analysis and HE staining. BMD was determined using a dual‐energy x‐ray absorptiometry scanner (DCS600EX‐IIIR, ALOKA Co. Ltd., Tokyo, Japan). Cone beam scanning was employed with settings of 55 kV and 145 mA, and microCT was utilized to scan the proximal femur. Femurs were fixed in 4% paraformaldehyde for 24 h, decalcified in 10% EDTA, and embedded in paraffin. Coronal sections were made at 5 μm, stained with HE (Sigma), and observed using a microscope (Olympus BX 53 microscope).

### 2.15 IHC Staining

The femurs were fixed with 4% paraformaldehyde for a week, followed by decalcification using 10% EDTA. The procedure involved encasing samples in paraffin, slicing them into 5‐μm slices, removing paraffin from xylene, inhibiting their peroxidase activity using 0.3% H_2_O_2_ for 10 min at ambient temperature, and then mixing them with PBS that included 5% FBS and 0.3% Triton X‐100 for an hour at the same temperature. OCN (AOPm; ab93876) and PPARγ (AOPm; ab209350) antibodies were incubated overnight at 4°C. After secondary antibody detection (ab6720, Abcam) for 1 h at room temperature, the target signals were developed by DAB substrate (Vector Labs, CA, USA), re‐stained with hematoxylin for 2 min, and visualized using a microscope (Leica).

### 2.16 Data Analysis

The experimental data were statistically analyzed using SPSS20. Data were expressed as mean ± SD and evaluated using t‐test or one‐way analysis of variance. *p < 0.05 indicated that the difference was statistically significant.

## 3 Results

### 3.1 Characterization of BMSCs and BMSC‐Exos

**FIGURE 1:**
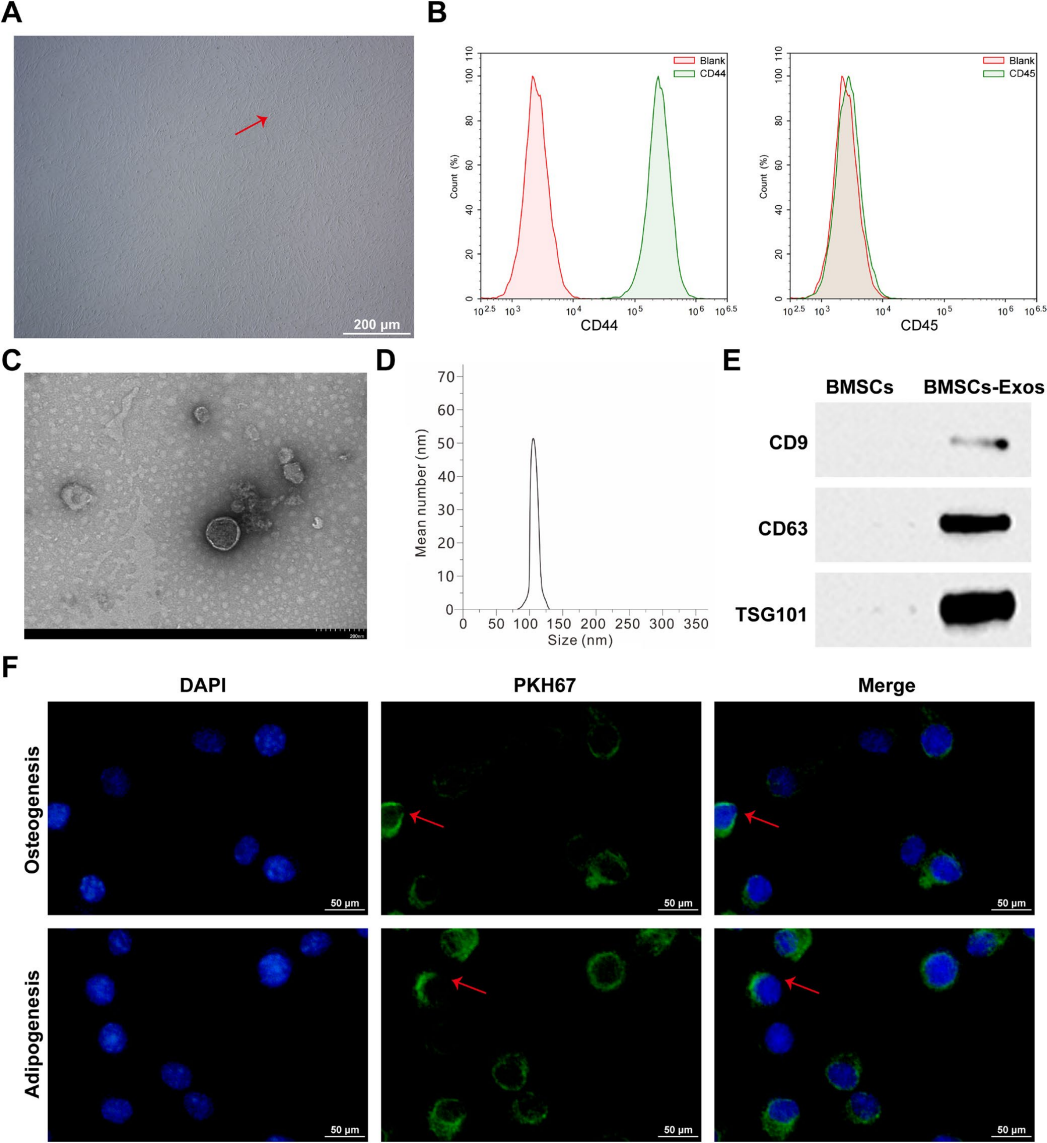
Identification of BMSCs and BMSC‐Exos. (A) TEM to observe the morphology of BMSCs extracted from the bone marrow cavity of mice. (B) Flow cytometry to determine the expression of surface markers of BMSCs. (C) TEM to observe the morphology of BMSC‐Exos. (D) NTA to measure the diameter distribution of BMSC‐Exos. (E) Western Blot to determine the expression levels of CD9, CD63, and TSG101 in BMSC‐Exos. (F) BMSCs osteoclasts and adipogenic cell uptake of BMSC‐Exos. Data are expressed as mean ± SD (*n* = 3). *p < 0.01.

The extracted BMSCs had a spindle‐shaped morphology with clear boundaries and strong refractive index (Figure 1A). Detection of BMSC surface antigens by flow cytometry showed that CD44 was positively expressed in the cells and CD45 was negatively expressed, indicating that the purity of the isolated BMSCs was high (Figure 1B). Observation by TEM showed that most of the BMSC‐Exos had a typical spherical bimodal structure with a diameter of about 80–130 nm (Figure 1C,D). Results determined by Western Blot showed that CD9, CD63, and TSG101 expression increased in BMSC‐Exos (Figure 1E). In addition, PKH67‐stained BMSC‐Exos showed green fluorescence, and DAPI‐stained BMSCs induced blue fluorescence in the nuclei of osteoblasts and adipocytes, as observed by fluorescence microscopy, suggesting that BMSC‐Exos have membrane fusion ability and can be taken up by osteoblasts and adipocytes (Figure 1F).

### 3.2 BMSC‐Exos Promote Osteogenic‐Adipogenic Balance of BMSCs

**FIGURE 2:**
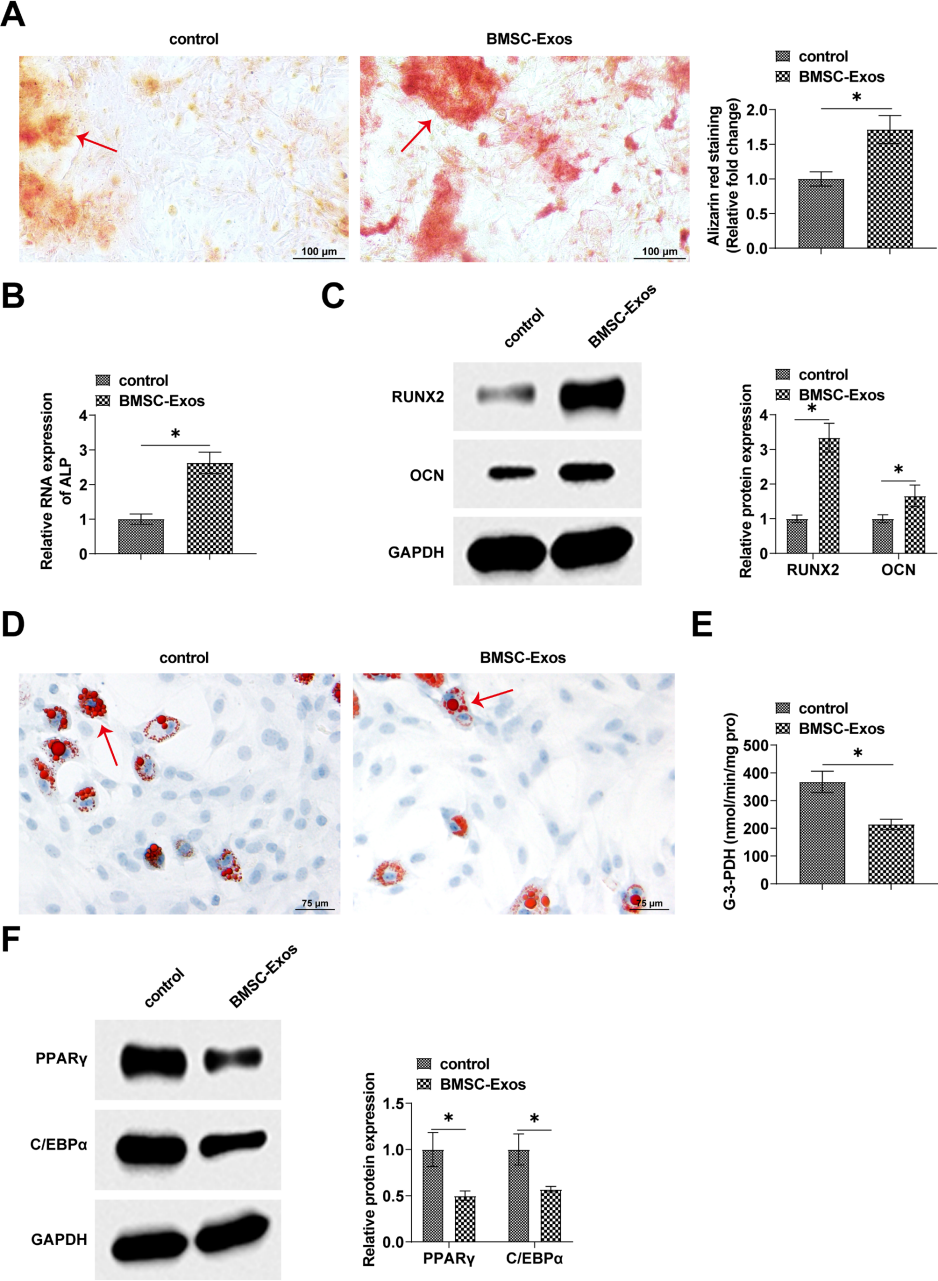
BMSC‐Exos promote osteogenic‐adipogenic balance in BMSCs. (A) Alizarin S red staining to detect calcium deposition in osteogenic differentiation of BMSCs. (B) RT‐qPCR to detect ALP level in BMSCs osteoblasts. (C) Western Blot to detect the expression of RUNX2 and OCN in BMSCs osteoblasts. (D) Oil red O staining to detect the formation of lipid droplets in BMSCs adipose cells. (E) Detection of G‐3‐PDH activity. (F) Western Blot to detect the expression of PPARγ and C/EBPα in BMSCs. Data are expressed as mean ± SD (*n* = 3). *p < 0.05.

BMSCs were induced into osteoblasts and adipose cells, and then co‐cultured with BMSC‐Exos. Alizarin S red staining showed that the staining of calcium deposition in osteoblasts was significantly larger after BMSC‐Exos treatment (Figure 2A). Meanwhile, RT‐qPCR assay showed that ALP levels were increased in BMSC‐Exos‐treated osteoblasts (Figure 2B). Western Blot assay showed that RUNX2 and OCN, proteins related to osteogenic differentiation, were upregulated in BMSCs after incubation with BMSC‐Exos (Figure 2C). Oil red O staining showed that lipid droplet formation was reduced in adipose cells after BMSC‐Exos treatment (Figure 2D). As shown in Figure 2E, BMSC‐Exos inhibited G‐3‐PDH activity, indicating that BMSC‐Exos inhibits adipogenesis. Also, Western Blot assay showed that BMSC‐Exos inhibited the up‐regulation of adipogenic differentiation‐related proteins PPARγ and C/EBPα (Figure 2F).

### 3.3 SNHG14 Enhances the Promoting Effect of BMSCs‐Exos on the Balance of Osteogenesis and Adipogenesis of BMSCs

**FIGURE 3:**
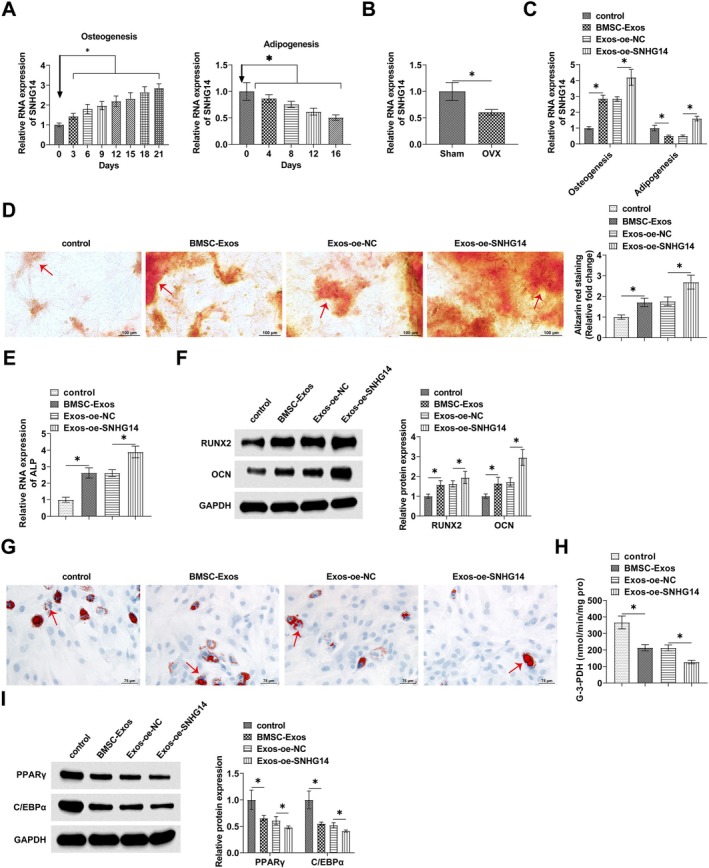
SNHG14 up‐regulation enhances the promotion of osteogenic‐adipogenic balance of BMSCs by BMSCs‐Exos. oe‐SNHG14 or oe‐NC was transfected into BMSCs. (A) RT‐qPCR to detect SNHG14 expression in BMSCs during osteogenic and adipogenic differentiation. (B) RT‐qPCR to detect SNHG14 expression in the femur tissue of OP mice. (C) RT‐qPCR to detect SNHG14 expression in osteoblasts and adipose cells treated with BMSC‐Exos overexpressing SNHG14. (D) Alizarin S red staining to detect calcium deposition in BMSCs. (E) RT‐qPCR to detect ALP levels in BMSCs osteoblasts. (F) Western Blot to detect the expression of RUNX2 and OCN in BMSCs. (G) Oil red O staining to detect lipid droplet formation in BMSCs. (H) Effect of oe‐SNHG14 on G‐3‐PDH activity. (I) Western Blot to detect the expression of PPARγ and C/EBPα in BMSCs. Data are expressed as mean ± SD (*n* = 3). *p < 0.05.

oe‐SNHG14 or oe‐NC was transfected into BMSCs, and BMSCs‐Exos were isolated and co‐cultured with BMSCs. SNHG14 in osteoblasts and adipose cells was detected by RT‐qPCR, and the results showed that SNHG14 expression was gradually up‐regulated during osteogenic differentiation of BMSCs, but gradually decreased during adipogenic differentiation (Figure 3A). Meanwhile SNHG14 expression was down‐regulated in the femurs of OP mice (Figure 3B). Oe‐SNHG14 in BMSC‐Exos significantly promoted the upregulation of SNHG14 expression in osteoblasts and adipose cells (Figure 3C). oe‐SNHG14 enhanced the promotion of calcium deposition staining by BMSC‐Exos in BMSCs (Figure 3D). Meanwhile, ALP levels were significantly increased in BMSC‐Exos‐treated BMSCs, and oe‐SNHG14 further enhanced the effect of BMSC‐Exos on ALP levels (Figure 3E). oe‐SNHG14 enhanced the promoting effect of BMSC‐Exos on RUNX2 and OCN levels in BMSCs (Figure 3F). Lipid droplet formation in BMSCs was reduced after BMSC‐Exos treatment, and oe‐SNHG14 enhanced the effect of BMSC‐Exos (Figure 3G). oe‐SNHG14 further enhanced the inhibitory effect of BMSC‐Exos on G‐3‐PDH activity (Figure 3H), as well as PPARγ and C/EBPα levels (Figure 3I).

### 3.4 SNHG14 Directly Binds miR‐27a‐3p

**FIGURE 4:**
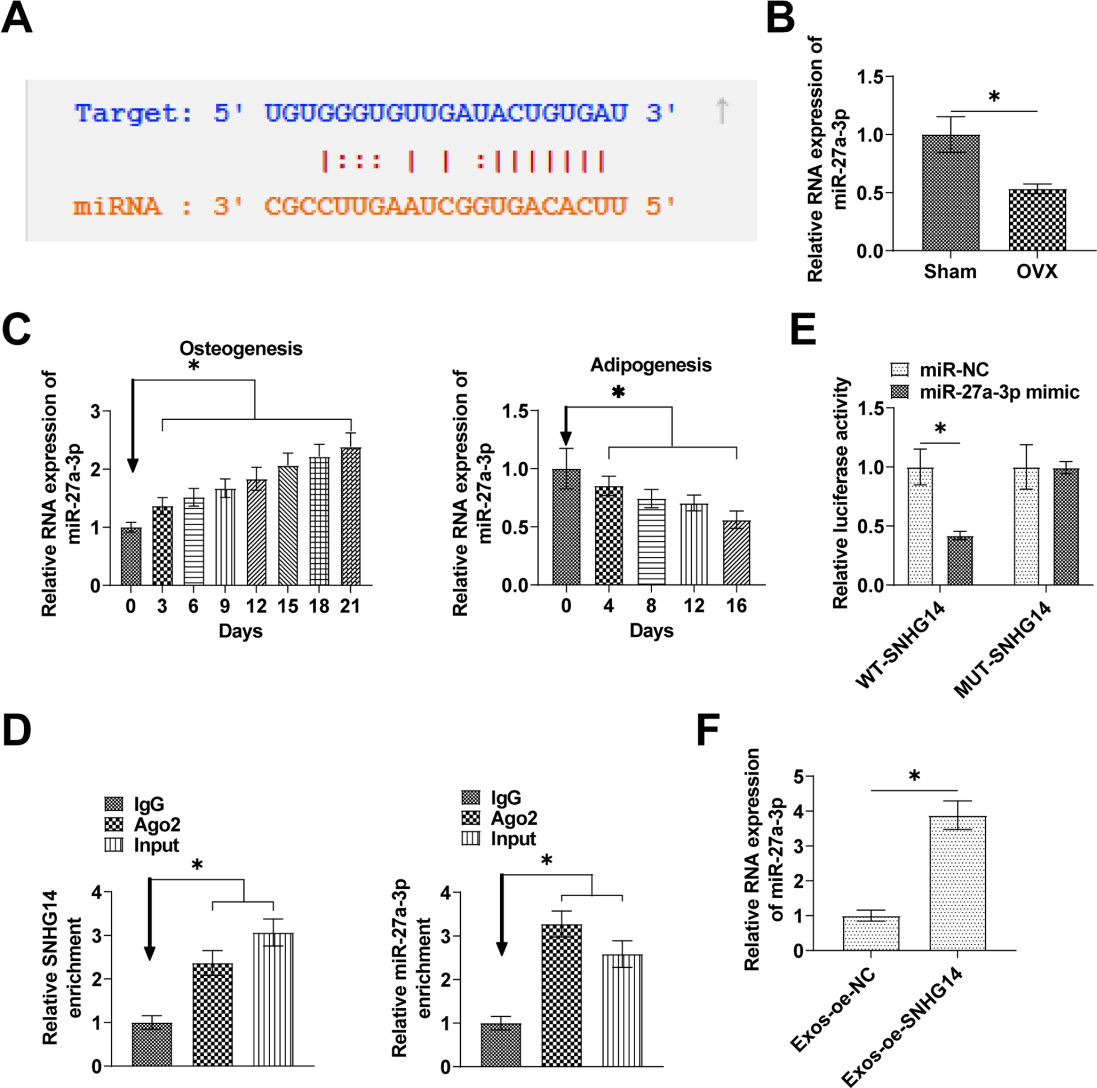
SNHG14 competitively binds miR‐27a‐3p. (A) StarBase to predict the binding site of miR‐27a‐3p to SNHG14. (B) RT‐qPCR to detect the expression of miR‐27a‐3p in OP tissues. (C) RT‐qPCR to detect the expression of miR‐27a‐3p in BMSCs during osteogenic and adipogenic differentiation. (D) RIP assay to detect the binding effect of SNHG14 to miR‐27a‐3p. (E) Dual luciferase reporter assay to detect the direct targeting relationship between miR‐27a‐3p and SNHG14. (F) RT‐qPCR assay to show the effect of oe‐SNHG14 on miR‐27a‐3p expression. Data are expressed as mean ± SD (*n* = 3). *p < 0.01.

Potential target miRNAs of SNHG14 were searched by the bioinformatics website starBase 3.0. Results confirmed a potential binding site for miR‐27a‐3p and SNHG14 (Figure 4A). RT‐qPCR observed that miR‐27a‐3p expression level in OP tissues was lower than that in normal tissues (Figure 4B). miR‐27a‐3p gradually increased as BMSCs were induced into osteoblasts, on the contrary, decreased and maintained at a low level throughout the process of adipogenic differentiation (Figure 4C). RIP assay experiments showed that both SNHG14 and miR‐27a‐3p were highly enriched in Ago2 (Figure 4D). Co‐transfecting miR‐27a‐3p mimic and WT‐SNHG14 with miR‐27a‐3p mimic resulted in a significant decrease in luciferase activity (Figure 4E). RT‐qPCR assay detected that oe‐SNHG14 increased miR‐27a‐3p expression (Figure 4F).

### 3.5 SNHG1 Mediates Osteogenic‐Adipogenic Balance in BMSCs via miR‐27a‐3p

**FIGURE 5:**
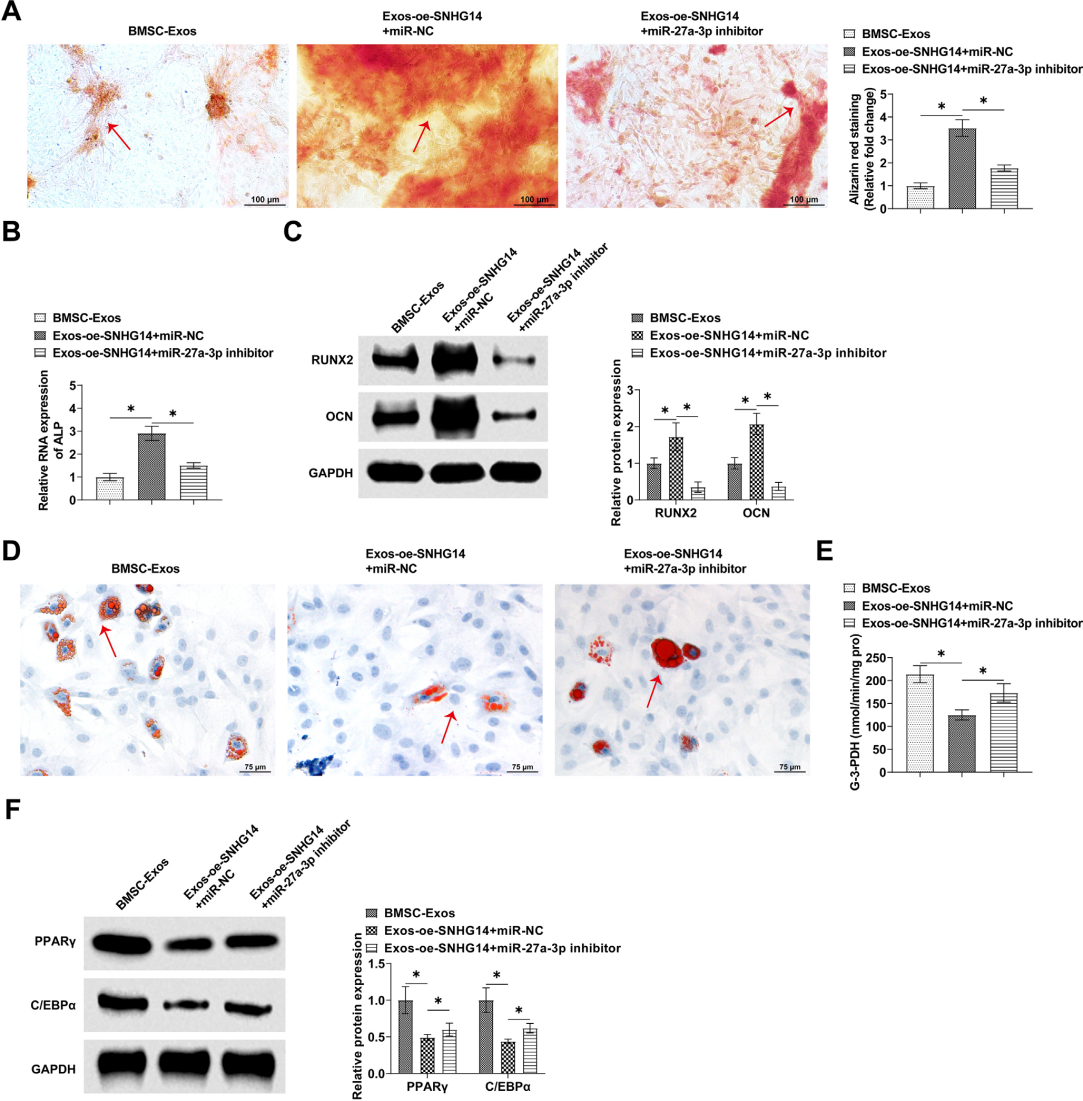
SNHG1 mediates osteogenic‐adipogenic balance in BMSCs via miR‐27a‐3p. miR‐27a‐3p inhibitor and oe‐SNHG14 were co‐transfected into BMSCs. (A) Alizarin S red staining to detect calcium deposition in BMSCs. (B) QRT‐PCR to detect ALP levels in BMSCs osteoblasts. (C) Western blot to detect the expression of RUNX2 and OCN in BMSCs. (D) Oil red O staining to detect the lipid droplet formation in BMSCs. (E) Effect of knockdown of miR‐27a‐3p on G‐3‐PDH activity. (F) Western Blot detection of PPARγ and C/EBPα expression in BMSCs. Data are expressed as mean ± SD (*n* = 3). *p < 0.05.

The interrelationship between SNHG14 and miR‐27a‐3p in BMSCs was explored by functional rescue experiments. miR‐27a‐3p inhibitor and oe‐SNHG14 were co‐transfected into BMSCs osteoblasts and adipocytes, and alizarin S red staining showed that oe‐SNHG14 enhanced the promotion of calcium deposition staining of BMSCs by BMSC‐Exos, whereas miR‐27a‐3p inhibitor reversed this effect (Figure 5A). Meanwhile, miR‐27a‐3p inhibitor reversed the promoting effect of oe‐SNHG14 on ALP levels (Figure 5B). Western Blot assay showed that oe‐SNHG14 promoted RUNX2 and OCN levels in BMSCs, while miR‐27a‐3p inhibitor reversed this effect (Figure 5C). Oil red O staining observed that oe‐SNHG14 treatment reduced the formation of lipid droplets in BMSCs, whereas miR‐27a‐3p inhibitor reversed the effect and promoted the formation of lipid droplets (Figure 5D). miR‐27a‐3p inhibitor treatment reversed the suppression of G‐3‐PDH activity and protein expressions of PPARγ and C/EBPα mediated by oe‐SNHG14 (Figure 5E,F).

### 3.6 LMNB1 Is the Target Gene of miR‐27a‐3p

**FIGURE 6:**
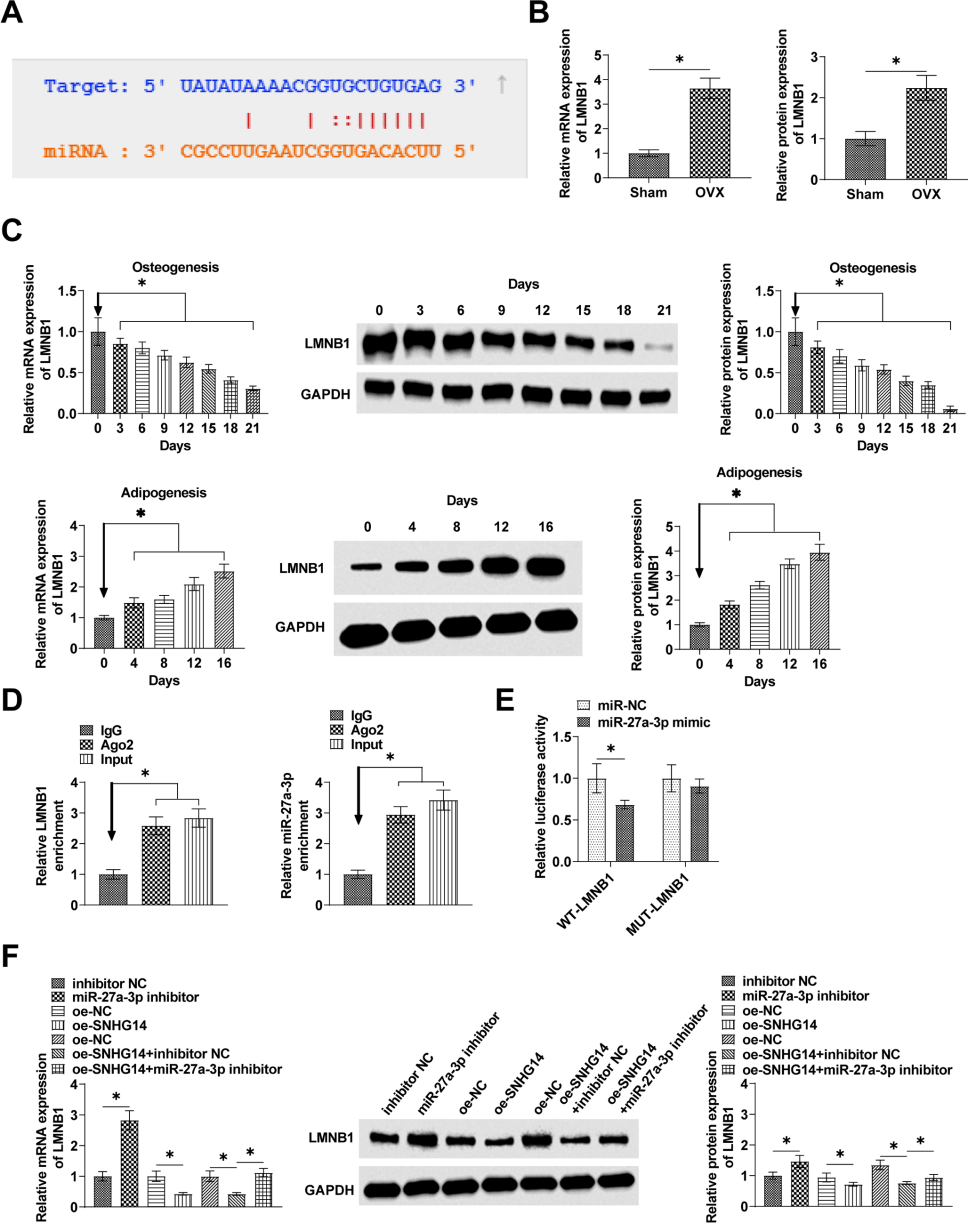
LMNB1 is the target gene of miR‐27a‐3p. (A) StarBase 3.0 to predict the binding site of miR‐27a‐3p and LMNB1. (B) RT‐qPCR and Western Blot to detect the expression of LMNB1 in the femur tissue of mice. (C) RT‐qPCR and Western Blot to detect the expression of LMNB1 in BMSCs. (D) RIP assay to determine the binding of miR‐27a‐3p to LMNB1. (E) Dual luciferase reporter assay to detect the targeting relationship between miR‐27a‐3p and LMNB1. (F) RT‐qPCR and Western Blot to detect LMNB1 expression in BMSCs. Data are expressed as mean ± SD (*n* = 3). *p < 0.01.

The molecular target of miR‐27a‐3p was found to be LMNB1 with possible binding sites predicted by the bioinformatics website starBase 3.0 (Figure 6A), and RT‐qPCR and Western Blot found high expression of LMNB1 in the femur tissue of OP mice (Figure 6B). The mRNA level of LMNB1 increased with BMSCs during adipogenic differentiation. Western blot showed that the protein level of LMNB1 was also upregulated during adipogenic differentiation. In contrast, both mRNA and protein levels of LMNB1 in BMSCs decreased and remained low throughout osteogenic differentiation (Figure 6C). LMNB1 and miR‐27a‐3p were shown to be significantly enriched in Ago2 immunomagnetic beads in RIP assay (Figure 6D). Luciferase activity was significantly inhibited by co‐transfecting miR‐27a‐3p mimic and WT‐LMNB1 in luciferase reporter gene assays (Figure 6E). miR‐27a‐3p inhibitor promoted LMNB1 levels in BMSCs; oe‐SNHG14 inhibited LMNB1 expression in BMSCs; miR‐27a‐3p inhibitor reversed the effect of oe‐SNHG14 on LMNB1 expression (Figure 6F).

### 3.7 LMNB1 Is Involved in the Process of SNHG14 Regulating Osteogenic‐Adipogenic Balance in BMSCs

**FIGURE 7:**
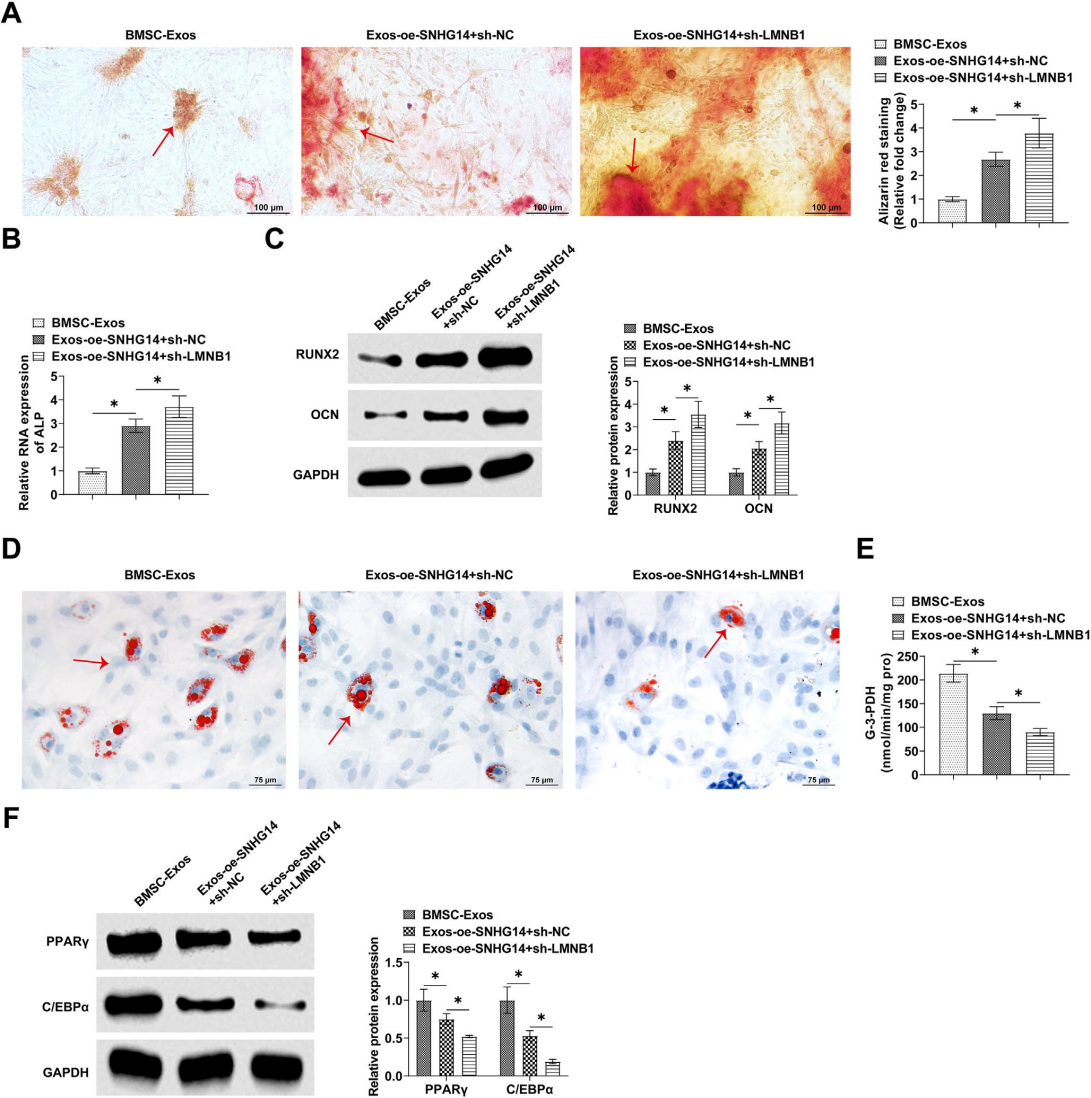
LMNB1 is involved in the process of SNHG14 regulating osteogenic‐adipogenic balance in BMSCs. sh‐LMNB1 and oe‐SNHG14 were co‐transfected into BMSCs. (A) Alizarin S red staining to detect calcium deposition in BMSCs. (B) QRT‐PCR to detect ALP levels in BMSCs osteoblasts. (C) Western blot to detect the expression of RUNX2 and OCN in BMSCs. (D) Oil red O staining to detect the lipid droplet formation in BMSCs. (E) Effect of knockdown of LMNB1 on G‐3‐PDH activity. (F) Western Blot detection of PPARγ and C/EBPα expression in BMSCs. Data are expressed as mean ± SD (*n* = 3). *p < 0.05.

After co‐transfection of sh‐LMNB1 and oe‐SNHG14 into BMSCs, it was recognized that sh‐LMNB1 enhanced the promotion of calcium deposition staining mediated by oe‐SNHG14 in BMSCs (Figure 7A). Meanwhile, sh‐LMNB1 promoted the facilitating effect of oe‐SNHG14 on ALP levels (Figure 7B). oe‐SNHG14 promoted RUNX2 and OCN mRNA in BMSCs, which was enhanced by sh‐LMNB1 (Figure 7C). Meanwhile, sh‐LMNB1 treatment reduced the formation of lipid droplets in BMSCs, promoting the action of oe‐SNHG14 (Figure 7D). sh‐LMNB1 significantly enhanced the effect of oe‐SNHG14 on G‐3‐PDH activity (Figure 7E) and protein expressions of PPARγ and C/EBPα (Figure 7F).

### 3.8 SNHG14 Overexpression Promotes Bone Formation and Alleviates OP In Vivo

**FIGURE 8:**
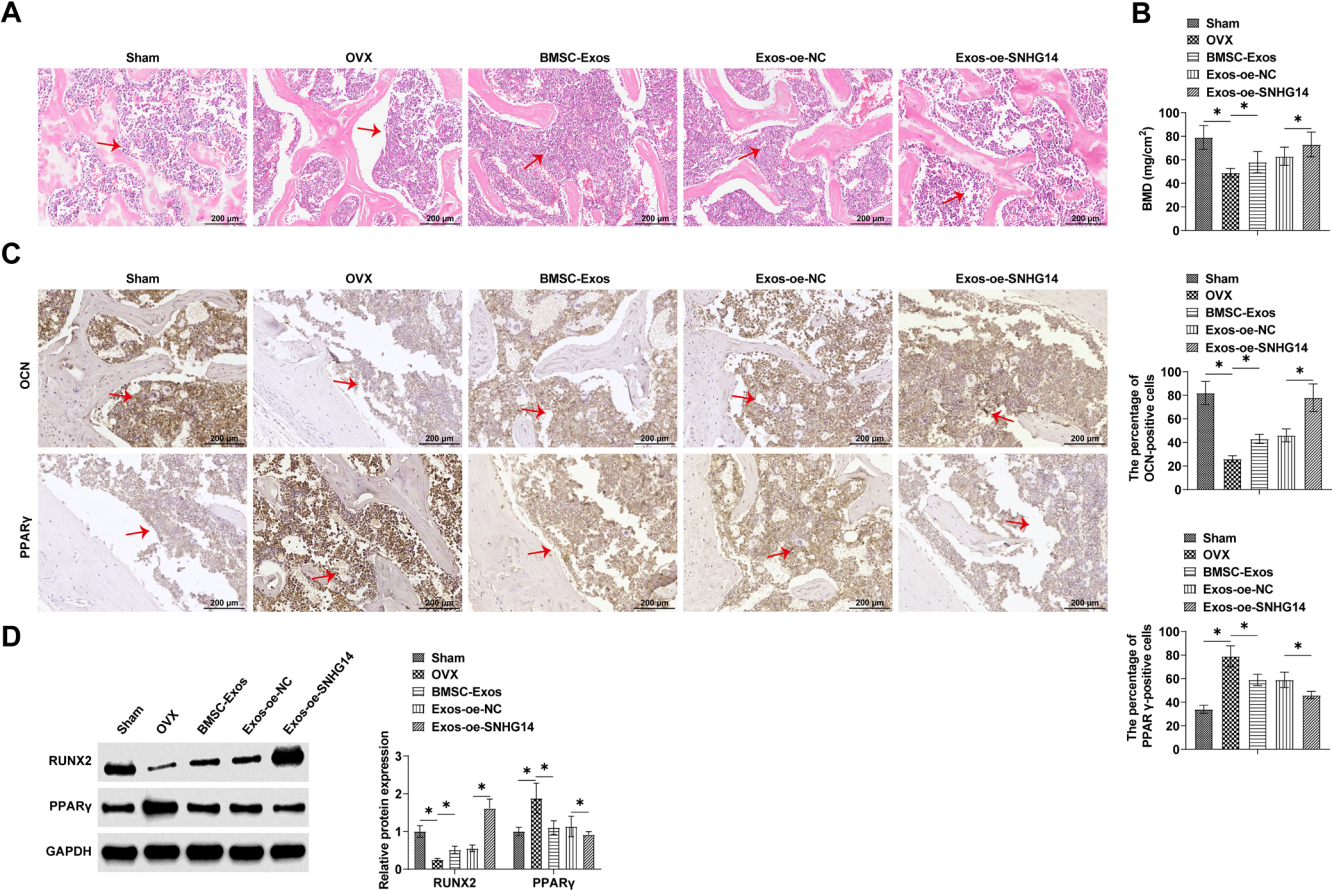
SNHG14 overexpression promotes bone formation and alleviates OP in vivo. (A) HE staining to assess bone tissue osteoblasts. (B) Measurement of BMD. (C) IHC to analyze the protein levels of osteogenic and adipogenic differentiation markers. (D) Western Blot to analyze the protein expression levels of RUNX2 and PPARγ. Data are expressed as mean ± SD (*n* = 3). *p < 0.05.

HE staining showed that osteoblasts were significantly reduced in OVX mice, but oe‐SNHG14 and BMSC‐Exos reversed this trend (Figure 8A). To test the osteogenesis of mice in each group, the BMD of mice was examined along with the protein expression with other bone formation markers (RUNX2 and OCN). The results analyzed by t‐test showed that the BMD of Sham mice was 79.00 ± 10.12 mg/cm^2^, and the BMD was significantly reduced to 49.00 ± 3.66 mg/cm^2^ after OVX surgery, which was significantly different (*p* < 0.05). This was consistent with the results of Western Blot and IHC for bone formation markers, which showed a significant down‐regulation of RUNX2 and OCN expression in OVX mice compared to Sham mice (*p* < 0.05). However, all the effects of OVX surgery were reversed by oe‐SNHG14 and BMSC‐Exos, and BMD was significantly elevated to 58.00 ± 9.01 mg/cm^2^ and 73.00 ± 10.55 mg/cm^2^ in mice, and the expression of RUNX2 and OCN was also significantly upregulated. oe‐SNHG14 treatment enhanced the effect of BMSC‐Exos, reducing PPARγ protein levels (*p* < 0.05). Western blot results showed the same results (Figure 8B–D). These findings indicated that BMSC‐Exos promoted bone formation and alleviated OP, and SNHG14 overexpression enhanced BMSC‐Exos action.

## 4 Discussion

For OP, promising therapeutic approaches involve targeting skeletal stem cells and osteoblasts, which are effective at promoting bone formation [21]. The data in this study determined that SNHG14 targeted the miR‐27a‐3p/LMNB1 axis to regulate osteogenic and adipogenic differentiation of BMSCs, suggesting that SNHG14 may be a valid target for the treatment of OP.

Due to the vast therapeutic potential of stem cell‐derived exosomes, their function has attracted much attention [22]. BMSC‐Exos have various ameliorative effects on different types of diseased cells in the human body [23]. More importantly, BMSC‐Exos have generated discussion in the field of bone tissue repair and regeneration because of their potential to restore or enhance bone damage, improve dysfunction, and repair missing tissues [24]. For example, BMSCs‐Exos enhance the regenerative capacity of osteoblasts in rats with cranial bone defect [25]. For this reason, we believe it is necessary to explore the underlying molecular mechanisms. A rise in osteogenic differentiation of BMSC is usually accompanied by a decrease in adipogenic differentiation. This balance of bone‐fat tissue is referred to as bone‐fat balancing [8]. Our research revealed that BMSC‐Exos‐mediated elevation of SNHG14 significantly improved the differentiation of osteoblasts and ALP function in BMSCs, with BMSCs treated with BMSC‐Exos exhibiting enhanced osteogenic differentiation capabilities. Additionally, we confirmed the impact of BMSC‐Exos on the adipogenic differentiation of BMSCs. The aforementioned findings indicate that BMSC‐Exos may balance osteogenesis and adipogenesis, offering a viable treatment approach for bone loss disorders.

LncRNAs are involved in various biological processes such as stem cell differentiation. Some lncRNAs have been shown to regulate fracture healing [26]. SNHG14 is known to be downregulated in BMSCs from OP patients [27]. Similarly, inhibition of SNHG14 blocks the differentiation of BMSCs into osteoblasts [16]. Mineralized tissues contain a high level of ALP, a positive indicator of osteoblast differentiation, and RUNX2 and OCN are key osteoblast specific genes. Our results showed that oe‐SNHG14 promoted the bone formation of BMSCs, which was manifested by the increase of ALP level, mineralization degree and RUNX2 and OCN levels. At the same time, oe‐SNHG14 inhibited the adipogenesis of BMSCs. Combined with the findings of in vivo experiments, we revealed the potential of SNHG14 as a new therapeutic target for OP.

miRNAs play a crucial regulatory role in bone remodeling [17]. miR‐27a‐3p induces osteogenic differentiation in hBMSC by activating osteogenic genes [18]. miR‐27a downregulation induces adipogenic differentiation, whereas miR‐27a inhibits adipogenesis and exacerbates osteogenesis by regulating PPARγ [28]. It is suggested that miR‐27a‐3p may be closely related to OP, including the imbalance between adipogenic and osteogenic differentiation. Our experimental results preliminarily confirmed this conjecture, and silencing miR‐27a‐3p inhibited osteogenic differentiation activity, promoted lipid droplet formation, and reversed the effects of oe‐SNHG14 and BMSC‐Exos.

LMNB1 was a possible target with a binding target to miR‐27a‐3p. LMNB1 upregulation exhibits adipocyte differentiation [29]. Similarly in our study, suppressing LMNB1 enhanced the effects of oe‐SNHG14 and BMSC‐Exos, enhancing the ability of BMSCs to differentiate into osteoblasts but reducing the ability to differentiate into adipocytes, thereby inhibiting bone loss.

Taken together, BMSCs‐derived exosomal SNHG14 mediates LMNB1 expression by acting as a miR‐27a‐3p sponge to regulate osteogenesis and adipogenesis balance. The findings provide a broader understanding of the pathogenesis of OP as well as new therapeutic strategies for treating it. However, further clinical trials are still needed to validate SNHG14 as a novel biomarker for OP in the future.

## Ethics Statement

The animal experiment research protocol was approved by the Ethics Committee of Huai'an Second People's Hospital (No. 2022‐HA313) and performed in accordance with the “Guidelines for the care and use of experimental animals.”

## Conflicts of Interest

The authors declare no conflicts of interest.

## Data Availability

The data that support the findings of this study are available from the corresponding author upon reasonable request.
